# One-way Resynchronizability of Word Transducers

**DOI:** 10.1007/978-3-030-71995-1_7

**Published:** 2021-03-23

**Authors:** Sougata Bose, S. N. Krishna, Anca Muscholl, Gabriele Puppis

**Affiliations:** 8 University of Oxford, Oxford, UK; 9 Sorbonne Université - LIP6, Paris, France; 10 LaBRI, University of Bordeaux, Bordeaux, France; 11 Dept. of Computer Science & Engineering, IIT Bombay, Bombay, India; 12 Dept. of Mathematics, Computer Science, and Physics, Univ. of Udine, Udine, Italy

**Keywords:** String transducers, Resynchronizers, One-way transducers

## Abstract

The origin semantics for transducers was proposed in 2014, and it led to various characterizations and decidability results that are in contrast with the classical semantics. In this paper we add a further decidability result for characterizing transducers that are close to one-way transducers in the origin semantics. We show that it is decidable whether a non-deterministic two-way word transducer can be resynchronized by a bounded, regular resynchronizer into an origin-equivalent one-way transducer. The result is in contrast with the usual semantics, where it is undecidable to know if a non-deterministic two-way transducer is equivalent to some one-way transducer.

## 1 Introduction

Regular word-to-word functions form a robust and expressive class of transformations, as they correspond to deterministic two-way transducers, to deterministic streaming string transducers [1], and to monadic second-order logical transductions [11]. However, the transition from word languages to functions over words is often quite tricky. One of the challenges is to come up with effective characterizations of restricted transformations. A first example is the characterization of functions computed by one-way transducers (known as *rational functions*). It turns out that it is decidable whether a regular function is rational [14], but the algorithm is quite involved [3]. In addition, non-determinism makes the problem intractable: it is undecidable whether the relation computed by a non-deterministic two-way transducer can be also computed by a one-way transducer, [2]. A second example is the problem of knowing whether a regular word function can be described by a first-order logical transduction. This question is still open in general [16], and it is only known how to decide if a *rational* function is definable in first-order logic [13].

Word transducers with origin semantics were introduced by Bojańczyk [4] and shown to provide a machine-independent characterization of regular word-

⋆ Work supported by ANR DeLTA (ANR-16-CE40-0007) and ReLaX.

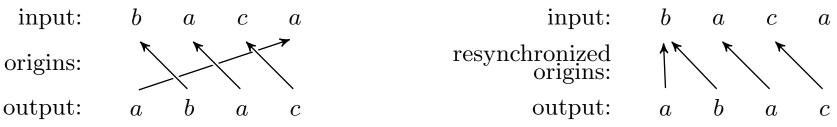

Fig. 1: On the left, an input-output pair for a transducer $T$ that reads $wd$ and outputs $dw$, $d \in \Sigma$, $w \in \Sigma^*$, the arrows denoting origins. On the right, the same input-output pair, but with origins modified by a resynchronizer $\mathcal{R}$. The resynchronized relation $\mathcal{R}(T)$ is order-preserving, and $T$ is one-way resynchronizable.

to-word functions. The origin semantics, as the name suggests, means tagging the output by the positions of the input that generated that output.

A nice phenomenon is that origins can restore decidability for some interesting problems. For example, the equivalence of word relations computed by one-way transducers, which is undecidable in the classical semantics [18,19], is PSPACE-complete for two-way non-deterministic transducers in the origin semantics [7]. Another, deeper, observation is that the origin semantics provides an algebraic approach that can be used to decide fragments. For example, [4] provides an effective characterization of first-order definable word functions under the origin semantics. As for the problem of knowing whether a regular word function is rational, it becomes almost trivial in the origin semantics.

A possible objection against the origin semantics is that the comparison of two transducers in the origin semantics is too strict. Resynchronizations were proposed in order to overcome this issue. A resynchronization is a binary relation between input-output pairs with origins, that preserves the input and the output, changing only the origins. Resynchronizations were introduced for one-way transducers [15], and later for two-way transducers [7]. For one-way transducers *rational* resynchronizations are transducers acting on the synchronization languages, whereas for two-way transducers, *regular* resynchronizations are described by regular properties over the input that restrict the change of origins. The class of bounded[4] regular resynchronizations was shown to behave very nicely, preserving the class of transductions defined by non-deterministic, two-way transducers: for any bounded regular resynchronization $\mathcal{R}$ and any two-way transducer $T$, the resynchronized relation $\mathcal{R}(T)$ can be computed by another two-way transducer [7]. In particular, non-deterministic, two-way transducers can be effectively compared modulo bounded regular resynchronizations.

As mentioned above, it is easy to know if a two-way transducer is equivalent under the origin semantics to some one-way transducer [4], since this is equivalent to being order-preserving. But what happens if this is not the case? Still, the given transducer $T$ can be "close" to some order-preserving transducer. What we mean here by "close" is that there exists some bounded regular resyn-

[4] "Bounded" refers here to the number of source positions that are mapped to the same target position. It rules out resynchronizations such as the universal one.

chronizer $\mathcal{R}$ such that $\mathcal{R}(T)$ is order-preserving and all input-output pairs with origins produced by $T$ are in the domain of $\mathcal{R}$. We call such transducers *one-way resynchronizable*. Figure 1 gives an example.

In this paper we show that it is decidable if a two-way transducer is one-way resynchronizable. We first solve the problem for bounded-visit two-way transducers. A bounded-visit transducer is one for which there is a uniform bound for the number of visits of any input position. Then, we use the previous result to show that one-way resynchronizability is decidable for arbitrary two-way transducers, so without the bounded-visit restriction. This is done by constructing, if possible, a bounded, regular resynchronization from the given transducer to a bounded-visit transducer with regular language outputs. Finally, we show that bounded regular resynchronizations are closed under composition, and this allows to combine the previous construction with our decidability result for bounded-visit transducers.

*Related work and paper overview.* The synthesis problem for resynchronizers asks to compute a resynchronizer from one transducer to another one, when the two transducers are given as input. The problem was studied in [6] and shown to be decidable for unambiguous two-way transducers (it is open for unrestricted transducers). The paper [21] shows that the containment version of the above problem is undecidable for unrestricted one-way transducers.

The origin semantics for streaming string transducers (SST) [1] has been studied in [5], providing a machine-independent characterization of the sets of origin graphs generated by SSTs. An open problem here is to characterize origin graphs generated by aperiodic streaming string transducers [10,16]. Going beyond words, [17] investigates decision problems of tree transducers with origin, and regains the decidability of the equivalence problem for non-deterministic top-down and MSO transducers by considering the origin semantics. An open problem for tree transducers with origin is that of synthesizing resynchronizers as in the word case.

We will recall regular resynchronizations in Section 3. Section 4 provides the proof ingredients for the bounded-visit case, and the proof of decidability of one-way resynchronizability in the bounded-visit case can be found in Section 5. Finally, in Section 6 we sketch the proof in the general case. A full version of the paper is available at `https://arxiv.org/abs/2101.08011`.

## 2 Preliminaries

Let $\Sigma$ be a finite input alphabet. Given a word $w \in \Sigma^*$ of length $|w| = n$, a *position* is an element of its domain $\mathsf{dom}(w) = \{1, \ldots, n\}$. For every position $i$, $w(i)$ denotes the letter at that position. A *cut* of $w$ is any number from 1 to $|w| + 1$, so a cut identifies a position *between* two consecutive letters of the input. The cut $i = 1$ represents the position just before the first input letter, and $i = |w| + 1$ the position just after the last letter of $w$.

*Two-way transducers.* We use two-way transducers as defined in [3,6], with a slightly different presentation than in classical papers such as [22]. As usual for two-way machines, for any input $w \in \Sigma^*$, $w(0) = \vdash$ and $w(|w|+1) = \dashv$, where $\vdash, \dashv \notin \Sigma$ are special markers used as delimiters.

A *two-way transducer* (or just *transducer* from now on) is a tuple $T = (Q, \Sigma, \Gamma, \Delta, I, F)$, where $\Sigma, \Gamma$ are respectively the input and output alphabets, $Q = Q_{\prec} \uplus Q_{\succ}$ is the set of states, partitioned into left-reading states from $Q_{\prec}$ and right-reading states from $Q_{\succ}$, $I \subseteq Q_{\succ}$ is the set of initial states, $F \subseteq Q$ is the set of final states, and $\Delta \subseteq Q \times (\Sigma \uplus \{\vdash, \dashv\}) \times \Gamma^* \times Q$ is the finite transition relation. Left-reading states read the letter to the left, whereas right-reading states read the letter to the right. This partitioning will also determine the head movement during a transition, as explained below.

As usual, to define runs of transducers we first define configurations. Given a transducer $T$ and a word $w \in \Sigma^*$, a *configuration* of $T$ on $w$ is a state-cut pair $(q, i)$, with $q \in Q$ and $1 \le i \le |w|+1$. A configuration $(q, i)$, $1 \le i \le |w|+1$ means that the automaton is in state $q$ and its head is *between* the $(i-1)$-th and the $i$-th letter of $w$. The transitions that depart from a configuration $(q, i)$ and read $a$ are denoted $(q, i) \xrightarrow{a} (q', i')$, and must satisfy one of the following:
(1) $q \in Q_{\succ}$, $q' \in Q_{\succ}$, $a = w(i)$, $(q, a, v, q') \in \Delta$, and $i' = i+1$,
(2) $q \in Q_{\succ}$, $q' \in Q_{\prec}$, $a = w(i)$, $(q, a, v, q') \in \Delta$, and $i' = i$,
(3) $q \in Q_{\prec}$, $q' \in Q_{\succ}$, $a = w(i-1)$, $(q, a, v, q') \in \Delta$, and $i' = i$,
(4) $q \in Q_{\prec}$, $q' \in Q_{\prec}$, $a = w(i-1)$, $(q, a, v, q') \in \Delta$, and $i' = i-1$. When $T$ has only right-reading states (i.e. $Q_{\prec} = \emptyset$), its head can only move rightward. In this case we call $T$ a *one-way transducer.*

A *run* of $T$ on $w$ is a sequence $\rho = (q_1, i_1) \xrightarrow{a_{j_1}|v_1} (q_2, i_2) \xrightarrow{a_{j_2}|v_2} \cdots \xrightarrow{a_{j_m}|v_m} (q_{m+1}, i_{m+1})$ of configurations connected by transitions. Note that the positions $j_1, j_2, \ldots, j_m$ of letters do not need to be ordered from smaller to bigger, and can differ slightly (by $+1$ or $-1$) from the cuts $i_1, i_2, \ldots, i_{m+1}$, since cuts take values in between consecutive letters.

A configuration $(q, i)$ on $w$ is *initial* (resp. *final*) if $q \in I$ and $i = 1$ (resp. $q \in F$ and $i = |w|+1$). A run is *successful* if it starts with an initial configuration and ends with a final configuration. The *output* associated with a successful run $\rho$ as above is the word $v_1 v_2 \cdots v_m \in \Gamma^*$. A transducer $T$ defines a relation $[\![T]\!] \subseteq \Sigma^* \times \Gamma^*$ consisting of all the pairs $(u, v)$ such that $v$ is the output of some successful run $\rho$ of $T$ on $u$.

*Origin semantics.* In the origin semantics for transducers [4] the output is tagged with information about the position of the input where it was produced. If reading the $i$-th letter of the input we output $v$, then all letters of $v$ are tagged with $i$, and we say they have *origin* $i$. We use the notation $(v, i)$ for $v \in \Gamma^*$ to denote that all positions in the output word $v$ have origin $i$, and we view $(v, i)$ as word over the alphabet $\Gamma \times \mathbb{N}$. The outputs associated with a successful run $\rho = (q_1, i_1) \xrightarrow{b_1|v_1} (q_2, i_2) \xrightarrow{b_2|v_2} (q_3, i_3) \cdots \xrightarrow{b_m|v_m} (q_{m+1}, i_{m+1})$ in the origin semantics are the words of the form $\nu = (v_1, j_1)(v_2, j_2) \cdots (v_m, j_m)$ over $\Gamma \times \mathbb{N}$ where, for all $1 \le k \le m$, $j_k = i_k$ if $q_k \in Q_{\succ}$, and $j_k = i_k - 1$ if $q_k \in Q_{\prec}$. Under the origin semantics, the relation defined by $T$, denoted $[\![T]\!]_o$, is the set of pairs $\sigma = (u, \nu)$ —called *synchronized pairs*— such that $u \in \Sigma^*$ and $\nu \in (\Gamma \times \mathbb{N})^*$ is the output of some successful run on $u$.

Equivalently, a synchronized pair $(u, \nu)$ can be described as a triple $(u, v, \mathit{orig})$, where $v$ is the projection of $\nu$ on $\Gamma$, and $\mathit{orig} : \mathsf{dom}(v) \to \mathsf{dom}(u)$ associates with each position of $v$ its origin in $u$. So for $\nu = (v_1, j_1)(v_2, j_2) \cdots (v_m, j_m)$ as above, $v = v_1 \dots v_m$, and, for all positions $i$ s.t. $|v_1 \dots v_{k-1}| < i \leq |v_1 \dots v_k|$, we have $\mathit{orig}(i) = j_k$. Given two transducers $T_1, T_2$, we say they are *origin-equivalent* if $[\![T_1]\!]_o = [\![T_2]\!]_o$. Note that two transducers $T_1, T_2$ can be equivalent in the classical semantics, $[\![T_1]\!] = [\![T_2]\!]$, while they can have different origin semantics, so $[\![T_1]\!]_o \neq [\![T_2]\!]_o$.

*Bounded-visit transducers.* Let $k > 0$ be some integer, and $\rho$ some run of a two-way transducer $T$. We say that $\rho$ is *$k$-visit* if for every $i \geq 0$, it has at most $k$ occurrences of configurations from $Q \times \{i\}$. We call a transducer $T$ *$k$-visit* if for every $\sigma \in [\![T]\!]_o$ there is some successful, $k$-visit run $\rho$ of $T$ with output $\sigma$ (actually we should call the transducer $k$-visit *in the origin semantics*, but for simplicity we omit this). For example, the relation $\{(w, \overline{w}) \mid w \in \Sigma^*\}$, where $\overline{w}$ denotes the reverse of $w$, can be computed by a 3-visit transducer. A transducer is called *bounded-visit* if it is $k$-visit for some $k$.

*Common guess.* It is often useful to work with a variant of two-way transducers that can guess beforehand some annotation on the input and inspect it consistently when visiting portions of the input multiple times. This feature is called *common guess* [5], and strictly increases the expressive power of two-way transducers, including bounded-visit ones.

## 3 One-way resynchronizability

### 3.1 Regular resynchronizers

Resynchronizations are used to compare transductions in the origin semantics. A *resynchronization* is a binary relation $\mathcal{R} \subseteq (\Sigma^* \times (\Gamma \times \mathbb{N})^*)^2$ over synchronized pairs such that $(\sigma, \sigma') \in \mathcal{R}$ implies that $\sigma = (u, v, \mathit{orig})$ and $\sigma' = (u, v, \mathit{orig}')$ for some origin mappings $\mathit{orig}, \mathit{orig}' : \mathsf{dom}(v) \to \mathsf{dom}(u)$. In other words, a resynchronization will only change the origin mapping, but neither the input, nor the output. Given a relation $S \subseteq \Sigma^* \times (\Gamma \times \mathbb{N})^*$ with origins, the *resynchronized relation* $\mathcal{R}(S)$ is defined as $\mathcal{R}(S) = \{\sigma' \mid (\sigma, \sigma') \in \mathcal{R},\ \sigma \in S\}$. For a transducer $T$ we abbreviate $\mathcal{R}([\![T]\!]_o)$ by $\mathcal{R}(T)$. The typical use of a resynchronization $\mathcal{R}$ is to ask, given two transducers $T, T'$, whether $\mathcal{R}(T)$ and $T'$ are origin-equivalent.

Regular resynchronizers (originally called MSO resynchronizers) were introduced in [7] as a resynchronization mechanism that preserves definability by two-way transducers. They were inspired by MSO (monadic second-order) transductions [9,12] and they are formally defined as follows. A *regular resynchronizer* is a tuple $\mathcal{R} = (\overline{I}, \overline{O}, \mathsf{ipar}, \mathsf{opar}, (\mathsf{move}_\tau)_\tau, (\mathsf{next}_{\tau,\tau'})_{\tau,\tau'})$ consisting of

- some monadic parameters (colors) $\overline{I} = (I_1, \dots, I_m)$ and $\overline{O} = (O_1, \dots, O_n)$,
- MSO sentences $\mathsf{ipar}, \mathsf{opar}$, defining languages over expanded input and output alphabets, i.e. over $\Sigma' = \Sigma \times 2^{\{1,\dots,m\}}$ and $\Gamma' = \Gamma \times 2^{\{1,\dots,n\}}$, respectively,
- MSO formulas $\mathsf{move}_\tau(y, z)$, $\mathsf{next}_{\tau,\tau'}(z, z')$ with two free first-order variables and parametrized by expanded output letters $\tau, \tau'$ (called types, see below).

To apply a regular resynchronizer as above, one first guesses the valuation of all the predicates $I_j, O_k$, and uses it to interpret the parameters $\overline{I}$ and $\overline{O}$. Based on the chosen valuation of the parameters $\overline{O}$, each position $x$ of the output $v$ gets an associated *type* $\tau_x = (v(x), b_1, \dots, b_n) \in \Gamma \times \{0,1\}^n$, where $b_j$ is 1 or 0 depending on whether $x \in O_j$ or not. We refer to the output word together with the valuation of the output parameters as *annotated output*, so a word over $\Gamma \times \{0,1\}^n$. Similarly, the *annotated input* is a word over $\Sigma \times \{0,1\}^m$. The annotated input and output word must satisfy the formulas $\mathsf{ipar}$ and $\mathsf{opar}$, respectively.

The origins of output positions are constrained using the formulas $\mathsf{move}_\tau$ and $\mathsf{next}_{\tau,\tau'}$, which are *parametrized by output types and evaluated over the annotated input.* Intuitively, the formula $\mathsf{move}_\tau(y, z)$ states how the origin of every output position of type $\tau$ changes from $y$ to $z$. We refer to $y$ and $z$ as *source* and *target* origin, respectively. The formula $\mathsf{next}_{\tau,\tau'}(z, z')$ instead constrains the target origins $z, z'$ of any two consecutive output positions with types $\tau$ and $\tau'$, respectively.

Formally, $\mathcal{R} = (\overline{I}, \overline{O}, \mathsf{ipar}, \mathsf{opar}, (\mathsf{move}_\tau), (\mathsf{next}_{\tau,\tau'}))$ defines the resynchronization consisting of all pairs $(\sigma, \sigma')$, with $\sigma = (u, v, \mathit{orig})$, $\sigma' = (u, v, \mathit{orig}')$, $u \in \Sigma^*$, and $v \in \Gamma^*$, for which there exist $u' \in \Sigma'^*$ and $v' \in \Gamma'^*$ such that

- $\pi_\Sigma(u') = u$ and $\pi_\Gamma(v') = v$
- $u'$ satisfies $\mathsf{ipar}$ and $v'$ satisfies $\mathsf{opar}$,
- $(u', \mathit{orig}(x), \mathit{orig}'(x))$ satisfies $\mathsf{move}_\tau$ for all $\tau$-labeled output positions $x \in \mathsf{dom}(v')$, and
- $(u', \mathit{orig}'(x), \mathit{orig}'(x{+}1))$ satisfies $\mathsf{next}_{\tau,\tau'}$ for all $x, x{+}1 \in \mathsf{dom}(v')$ such that $x$ and $x + 1$ have label $\tau$ and $\tau'$, respectively.

*Example 1.* Consider the following resynchronization $\mathcal{R}$. A pair $(\sigma, \sigma')$ belongs to $\mathcal{R}$ if $\sigma = (uv, uwv, \mathit{orig})$, $\sigma' = (uv, uwv, \mathit{orig}')$, with $u, v, w \in \Sigma^+$. The origins $\mathit{orig}$ and $\mathit{orig}'$ are both the identity over $u$ and $v$. The origin of every position of $w$ in $\sigma$ (hence a source origin) is either the first or the last position of $v$. The origin of every position of $w$ in $\sigma'$ (a target origin) is the first position of $v$.

This resynchronization is described by a regular resynchronizer that uses two input parameters $I_1, I_2$ to mark the last and the first positions of $v$ in the input, and one output parameter $O$ to mark the factor $w$ in the output. The formula $\mathsf{move}_\tau(y, z)$ is either $(I_1(y) \vee I_2(y)) \wedge I_2(z)$ or $(y = z)$, depending on whether the type $\tau$ describes a position inside $w$ or a position outside $w$.

We now turn to describing some important restrictions on (regular) resynchronizers. Let $\mathcal{R} = (\overline{I}, \overline{O}, \mathsf{ipar}, \mathsf{opar}, (\mathsf{move}_\tau), (\mathsf{next}_{\tau,\tau'}))$ be a resynchronizer.

- $\mathcal{R}$ is *k-bounded* (or just *bounded*) if for every annotated input $u' \in \Sigma'^*$, every output type $\tau \in \Gamma'$, and every position $z$, there are at most $k$ positions $y$ such that $(u', y, z)$ satisfies $\mathsf{move}_\tau$. Recall that $y, z$ are input positions.
- $\mathcal{R}$ is *T-preserving* for a given transducer $T$, if every $\sigma \in [\![T]\!]_o$ belongs to the domain of $\mathcal{R}$.
- $\mathcal{R}$ is *partially bijective* if each $\mathsf{move}_\tau$ formula defines a partial, bijective function from source origins to target origins. Observe that this property implies that $\mathcal{R}$ is 1-bounded.

The boundedness restriction rules out resynchronizations such as the universal one, that imposes no restriction on the change of origins. It is a decidable restriction [7], and it guarantees that definability by two-way transducers is effectively preserved under regular resynchronizations, modulo common guess. More precisely, Theorem 16 in [7] shows that, given a bounded regular resynchronizer $\mathcal{R}$ and a transducer $T$, one can construct a transducer $T'$ with common guess that is origin-equivalent to $\mathcal{R}(T)$.

*Example 1 (continued).* Consider again the regular resynchronizer $\mathcal{R}$ described in the previous example. Note that $\mathcal{R}$ is 2-bounded, since at most two source origins are redirected to the same target origin. If we used an additional output parameter to distinguish, among the positions of $w$, those that have source origin in the first position of $v$ and those that have source origin in the last position of $v$, we would get a 1-bounded, regular resynchronizer.

We state below two crucial properties of regular resynchronizers (the second lemma is reminiscent of Lemma 11 from [21], which proves closure of bounded resynchronizers with vacuous $\mathsf{next}_{\tau,\tau'}$ relations).

**Lemma 1.** *Every bounded, regular resynchronizer is effectively equivalent to some* 1*-bounded, regular resynchronizer.*

**Lemma 2.** *The class of bounded, regular resynchronizers is effectively closed under composition.*

### 3.2 Main result

Given a two-way transducer $T$ one can ask if it is origin-equivalent to some one-way transducer. It was observed in [4] that this property holds if and only if all synchronized pairs defined by $T$ are *order-preserving*, namely, for all $\sigma = (u, v, orig) \in [\![T]\!]_o$ and all $y, y' \in \mathsf{dom}(v)$, with $y < y'$, we have $orig(y) \leq orig(y')$. The decidability of the above question should be contrasted to the analogous question in the classical semantics: "is a given two-way transducer classically equivalent to some one-way transducer?" The latter problem turns out to be decidable for functional transducers [14,3], but is undecidable for arbitrary two-way transducers [2].

Here we are interested in a different, more relaxed notion:

**Definition 1.** *A transducer $T$ is called* one-way resynchronizable *if there exists a bounded, regular resynchronizer $\mathcal{R}$ that is $T$-preserving and such that $\mathcal{R}(T)$ is order-preserving.*

Note that if $T'$ is an order-preserving transducer, then one can construct rather easily a one-way transducer $T''$ such that $T' =_o T''$, by eliminating non-productive U-turns from accepting runs.

Moreover, note that without the condition of being $T$-preserving every transducer $T$ would be one-way resynchronizable, using the empty resynchronization.

*Example 2.* Consider the transducer $T_1$ that moves the last letter of the input $wa$ to the front by a first left-to-right pass that outputs the last letter $a$, followed by a right-to-left pass without output, and finally by a left-to-right pass that produces the remaining $w$. Let $\mathcal{R}$ be the bounded regular resynchronizer that redirects the origin of the last $a$ to the first position. Assuming an output parameter $O$ with an interpretation constrained by $\mathsf{opar}$ that marks the last position of the output, the formula $\mathsf{move}_{(a,1)}(y,z)$ says that target origin $z$ (source origin $y$, resp.) of the last $a$ is the first (last, resp.) position of the input. It is easy to see that $\mathcal{R}(T_1)$ is origin-equivalent to the one-way transducer that on input $wa$, guesses $a$ and outputs $aw$. Thus, $T_1$ is one-way resynchronizable. See also Figure 1.

*Example 3.* Consider the transducer $T_2$ that reads inputs of the form $u\#v$ and outputs $vu$ in the obvious way, by a first left-to-right pass that outputs $v$, followed by a right-to-left pass, and a finally a left-to-right pass that outputs $u$. Using the characterization with the notion of cross-width that we introduce below, it can be shown that $T_2$ is not one-way resynchronizable.

In order to give a flavor of our results, we anticipate here the two main theorems, before introducing the key technical concepts of cross-width and inversion (these will be defined further below).

**Theorem 1.** *For every bounded-visit transducer $T$, the following are equivalent:*

*(1) $T$ is one-way resynchronizable,*
*(2) the cross-width of $T$ is finite,*
*(3) no successful run of $T$ has inversions,*
*(4) there is a partially bijective, regular resynchronizer $\mathcal{R}$ that is $T$-preserving and such that $\mathcal{R}(T)$ is order-preserving.*

*Moreover, condition (3) is decidable.*

We will use Theorem 1 to show that one-way resynchronizability is decidable for arbitrary two-way transducers (not just bounded-visit ones).

**Theorem 2.** *It is decidable whether a given two-way transducer $T$ is one-way resynchronizable.*

Let us now introduce the first key concept, that of cross-width:

**Definition 2 (cross-width).** *Let* $\sigma = (u, v, orig)$ *be a synchronized pair and let* $X_1, X_2 \subseteq \mathsf{dom}(v)$ *be sets of output positions such that, for all* $x_1 \in X_1$ *and* $x_2 \in X_2$, $x_1 < x_2$ *and* $orig(x_1) > orig(x_2)$. *We call such a pair* $(X_1, X_2)$ *a* cross *and define its* width *as* $\min(|orig(X_1)|, |orig(X_2)|)$, *where* $orig(X) = \{orig(x) \mid x \in X\}$ *is the set of origins corresponding to a set* $X$ *of output positions. The* cross-width *of a synchronized pair* $\sigma$ *is the maximal width of the crosses in* $\sigma$. *A transducer has* bounded cross-width *if for some integer* $k$, *all synchronized pairs associated with successful runs of* $T$ *have cross-width at most* $k$.

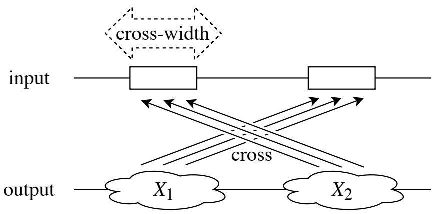

For instance, the transducer $T_2$ in Example 3 has unbounded cross-width. In contrast, the transducer $T_1$ in Example 2 has cross-width one.

The other key notion of *inversion* will be introduced formally in the next section (page 135), as it requires a few technical definitions. The notion however is very similar in spirit to that of cross, with the difference that a single inversion is sufficient for witnessing a family of crosses with arbitrarily large cross-width.

## 4 Proof overview for Theorem 1

This section provides an overview of the proof of Theorem 1, and introduces the main ingredients.

We will use flows (a concept inspired from crossing sequences [22,3] and revised in Section 4.1) in order to derive the key notion of inversion. Roughly speaking, an inversion in a run involves two loops that produce outputs in an order that is reversed compared to the order on origins. Inversions were also used in the characterization of one-way definability of two-way transducers under the classical semantics [3]. There, they were used for deriving some combinatorial properties of outputs. Here we are only interested in detecting inversions, and this is a simple task.

Flows will also be used to associate factorization trees with runs (the existence of factorization trees of bounded height was established by the celebrated Simon's factorization theorem [23]). We will use a structural induction on these factorization trees and the assumption that there is no inversion in every run to construct a regular resynchronization witnessing one-way resynchronizability of the transducer at hand.

Another important ingredient underlying the main characterization is given by the notion of dominant output interval (Section 4.2), which is used to formalize the invariant of our inductive construction.

### 4.1 Flows and inversions

*Intervals.* An *interval* of a word is a set of consecutive positions in it. An interval is often denoted by $I = [i, i')$, with $i = \min(I)$ and $i' = \max(I) + 1$. Given two intervals $I = [i, i')$ and $J = [j, j')$, we write $I < J$ if $i' \leq j$, and we say that $I, J$ are adjacent if $i' = j$. The union of two adjacent intervals $I = [i, i')$, $J = [j, j')$, denoted $I \cdot J$, is the interval $[i, j')$ (if $I, J$ are not adjacent, then $I \cdot J$ is undefined).

*Subruns.* Given a run $\rho$ of a transducer, a *subrun* is a factor of $\rho$. Note that a subrun of a two-way transducer may visit a position of the input several times. For an input interval $I = [i, j)$ and a run $\rho$, we say that a subrun $\rho'$ of $\rho$ *spans over* $I$ if $i$ (resp. $j$) is the smallest (resp. greatest) input position labeling some transition of $\rho'$. The left hand-side of the figure at page 134 gives an example of an interval $I$ of an input word together with the subruns $\alpha_1, \alpha_2, \alpha_3, \beta_1, \beta_2, \beta_3, \gamma_1$ that span over it. Subruns spanning over an interval can be left-to-right, left-to-left, right-to-left, or right-to-right depending on where the starting and ending positions are w.r.t. the endpoints of the interval.

*Flows.* Flows are used to summarize subruns of a two-way transducer that span over a given interval. The definition below is essentially taken from [3], except for replacing "functional" by "$K$-visit". Formally, a *flow* of a transducer $T$ is a graph with vertices divided into two groups, L-vertices and R-vertices, labeled by states of $T$, and with directed edges also divided into two groups, productive and non-productive edges. The graph satisfies the following requirements. Edge sources are either an L-vertex labeled by a right-reading state, or an R-vertex labeled by a left-reading state, and symmetrically for edge destinations; moreover, edges are of one of the following types: LL, LR, RL, RR. Second, each node is the endpoint of exactly one edge. Finally, L (R, resp.) vertices are totally ordered, in such a way that for every LL (RR, resp.) edge $(v, v')$, we have $v < v'$. We will only consider flows of $K$-visiting transducers, so flows with at most $2K$ vertices. For example, the flow in the left-hand side of the figure at page 134 has six L-vertices on the left, and six R-vertices on the right. The edges $\alpha_1$, $\alpha_2$, $\alpha_3$ are LL, LR, and RR, respectively.

Given a run $\rho$ of $T$ and an interval $I = [i, i')$ on the input, *the flow of* $\rho$ *on* $I$, denoted $\mathit{flow}_\rho(I)$, is obtained by identifying every configuration at position $i$ (resp. $i'$) with an L (resp. R) vertex, labeled by the state of the configuration, and every subrun spanning over $I$ with an edge connecting the appropriate vertices (this subrun is called the *witnessing subrun* of the edge of the flow). An edge is said to be *productive* if its witnessing subrun produces non-empty output.

*Flow monoid.* The composition of two flows $F$ and $G$ is defined when the R-vertices of $F$ induce the same sequence of labels as the L-vertices of $G$. In this case, the composition results in the flow $F \cdot G$ that has as vertices the L-vertices of $F$ and the R-vertices of $G$, and for edges the directed paths in the graph obtained by glueing the R-vertices of $F$ with the L-vertices of $G$ so that states are matched. Productiveness of edges is inherited by paths, implying that an edge of $F \cdot G$ is productive if and only if the corresponding path contains at least one edge (from $F$ or $G$) that is productive. When the composition is undefined, we simply write $F \cdot G = \bot$. The above definitions naturally give rise to a *flow monoid* associated with the transducer $T$, where elements are the flows of $T$, extended with a dummy element $\perp$, and the product operation is given by the composition of flows, with the convention that $\perp$ is absorbing. It is easy to verify that for any two adjacent intervals $I < J$ of a run $\rho$, $\mathit{flow}_\rho(I) \cdot \mathit{flow}_\rho(J) = \mathit{flow}_\rho(I \cdot J)$. We denote by $M_T$ the *flow monoid* of a $K$-visiting transducer $T$.

Let us estimate the size of $M_T$. If $Q$ is the set of states of $T$, there are at most $|Q|^{2K}$ possible sequences of L and R-vertices; and the number of edges (marked as productive or not) is bounded by $\binom{2K}{K} \cdot (2K)^K \cdot 2^K \leq (2K+1)^{2K}$. Including the dummy element $\perp$ in the flow monoid, we get $|M_T| \leq (|Q| \cdot (2K+1))^{2K} + 1 =: \mathbf{M}$.

*Loops.* A loop of a run $\rho$ over input $w$ is an interval $I = [i, j)$ with a flow $F = \mathit{flow}_\rho(I)$ such that $F \cdot F = F$ (call $F$ *idempotent*). The run $\rho$ can be pumped on a loop $I = [i, j)$ as expected: given $n > 0$, we let $\mathit{pump}^n_I(\rho)$ be the run obtained from $\rho$ by glueing the subruns that span over the intervals $[1, i)$ and $[j, |w| + 1)$ with $n$ copies of the subruns spanning over $I$ (see figure to the right).

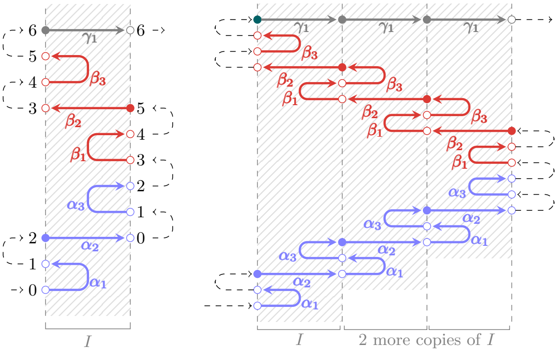

The lemma below shows that the occurrence order relative to subruns witnessing LR or RL edges of a loop (called *straight edges*, for short) is preserved when pumping the loop. This seemingly straightforward lemma is needed for detecting inversions and its proof is surprisingly non-trivial. For example, the external edge connecting the two L-vertices 1, 2 in the figure above appears before edge $\alpha_2$, and also before every copy of $\alpha_2$ in the run where loop $I$ is pumped.

**Lemma 3.** *Let $\rho$ be a run of $T$ on $u$, let $J < I < K$ be a partition of the domain of $u$ into intervals, with $I$ loop of $\rho$, and let $F = \mathit{flow}_\rho(J)$, $E = \mathit{flow}_\rho(I)$, and $G = \mathit{flow}_\rho(K)$ be the corresponding flows. Consider an arbitrary edge $f$ of either $F$ or $G$, and a straight edge $e$ of the idempotent flow $E$. Let $\rho_f$ and $\rho_e$ be the witnessing subruns of $f$ and $e$, respectively. Then the occurrence order of $\rho_f$ and $\rho_e$ in $\rho$ is the same as the occurrence order of $\rho_f$ and any copy of $\rho_e$ in $\mathit{pump}^n_I(\rho)$.*

We can now recall the key notion of inversion:

**Definition 3 (inversion).** *An* inversion *of $\rho$ is a tuple $(I, e, I', e')$ such that*

- *$I, I'$ are loops of $\rho$ and $I < I'$,*
- *$e, e'$ are productive straight edges in $flow_\rho(I)$ and $flow_\rho(I')$ respectively,*
- *the subrun witnessing $e'$ precedes the subrun witnessing $e$ in the run order*

*(see the figure to the right).*

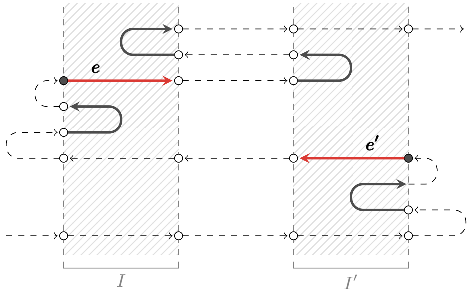

## 4.2 Dominant output intervals

In this section we identify some particular intervals of the output that play an important role in the inductive construction of the resynchronizer for a one-way resynchronizable transducer.

Given $n \in \mathbb{N}$, we say that a set $B$ of output positions is *n-large* if $|orig(B)| > n$; otherwise, we say that $B$ is *n-small.* Recall that here we work with a $K$-visiting transducer $T$, for some constant $K$, and that $\mathbf{M} = (|Q| \cdot (2K+1))^{2K} + 1$ is an upper bound to the size of the flow monoid $M_T$. We will extensively use the derived constant $\mathbf{C} = \mathbf{M}^{2K}$ to distinguish between large and small sets of output positions. The intuition behind this constant is that any set of output positions that is $\mathbf{C}$-large must traverse a loop of $\rho$. This is captured by the lemma below. The proof uses algebraic properties of the flow monoid $M_T$ [20] (see also Theorem 7.2 in [3], which proves a similar result, but with a larger constant derived from Simon's factorization theorem):

**Lemma 4.** *Let $I$ be an input interval and $B$ a set of output positions with origins inside $I$. If $B$ is $\mathbf{C}$-large, then there is a loop $J \subseteq I$ of $\rho$ such that $flow_\rho(J)$ contains a productive straight edge witnessed by a subrun that intersects $B$ (in particular, $out(J) \cap B \neq \emptyset$).*

We need some more notations for outputs. Given an input interval $I$ we denote by $out_\rho(I)$ the set of output positions whose origins belong to $I$ (note that this might not be an output interval). An *output block* of $I$ is a maximal interval contained in $out_\rho(I)$.

The *dominant output interval* of $I$, denoted $bigout_\rho(I)$, is the smallest output interval that contains all $\mathbf{C}$-large output blocks of $I$. In particular, $bigout_\rho(I)$ either is empty or begins with the first $\mathbf{C}$-large output block of $I$ and ends with the last $\mathbf{C}$-large outblock block of $I$. We will often omit the subscript $\rho$ from the notations $flow_\rho(I)$, $out_\rho(I)$, $bigout_\rho(I)$, etc., when no confusion arises.

We now fix a successful run $\rho$ of the $K$-visiting transducer $T$. The rest of the section presents some technical lemmas that will be used in the inductive constructions for the proof of the main theorem. *In the lemmas below, we assume that all successful runs of $T$ (in particular, $\rho$) avoid inversions.*

**Lemma 5.** *Let $I_1 < I_2$ be two input intervals and $B_1, B_2$ output blocks of $I_1$, $I_2$, respectively. If both $B_1, B_2$ are* **C***-large, then $B_1 < B_2$.*

*Proof (sketch).* If the claim would not hold, then Lemma 4 would provide some loops $J_1 \subseteq I_1$ and $J_2 \subseteq I_2$, together with some productive edges in them, witnessing an inversion. □

**Lemma 6.** *Let $I = I_1 \cdot I_2$, $B = bigout(I)$, and $B_i = bigout(I_i)$ for $i = 1, 2$. Then $B \setminus (B_1 \cup B_2)$ is $4K\mathbf{C}$-small.*

*Proof (sketch).* By Lemma 5, $B_1 < B_2$. Moreover, all **C**-large output blocks of $I_1$ or $I_2$ are also **C**-large output blocks of $I$, so $B$ contains both $B_1$ and $B_2$. Suppose, by way of contradiction, that $B \setminus (B_1 \cup B_2)$ is $4K\mathbf{C}$-large. This means that there is a $2K\mathbf{C}$-large set $S \subseteq B \setminus (B_1 \cup B_2)$ with origins entirely to the left of $I_2$, or entirely to the right of $I_1$. Suppose, w.l.o.g., that the former case holds, and decompose $S$ as a union of maximal output blocks $B'_1, B'_2, \ldots, B'_n$ with origins either entirely inside $I_1$, or entirely outside. Since $S \cap B_1 = \emptyset$, every block $B'_i$ with origins inside $I_1$ is **C**-small. Similarly, one can prove that every block $B'_i$ with origins outside $I_1$ is **C**-small too. Moreover, since $\rho$ is $K$-visiting, we get $n \leq 2K$. Altogether, this contradicts the assumption that $S$ is $2K\mathbf{C}$-large. □

**Lemma 7.** *Let $I = I_1 \cdot I_2 \cdots I_n$, such that $I$ is a loop and $flow(I) = flow(I_k)$ for all $k$. Then $bigout(I)$ can be decomposed as $B_1 \cdot J_1 \cdot B_2 \cdot J_2 \cdot \ldots \cdot J_{n-1} \cdot B_n$, where*

1. *for all $1 \leq k \leq n$, $B_k = bigout(I_k)$ (with $B_k$ possibly empty);*
2. *for all $1 \leq k < n$, the positions in $J_k$ have origins inside $I_k \cup I_{k+1}$ and $J_k$ is $2K\mathbf{C}$-small.*

*Proof (sketch).* The proof idea is similar to the previous lemma. First, using properties of idempotent flows, one shows that all output positions strictly between $B_k$ and $B_{k+1}$, for any $k = 1, \ldots, n-1$, have origin in $I_k \cup I_{k+1}$. Then, one observes that every output block of $I_k$ disjoint from $B_k$ is **C**-small, and since $T$ is $K$-visiting there are at most $K$ such blocks. This shows that every output interval $J_k$ between $B_k$ and $B_{k+1}$ is $2K\mathbf{C}$-small. For an illustration see the figure to the right. The **C**-large blocks in $I_1$ are shown in red; in blue those for $I_2$, in purple those for $I_3$. So $bigout(I_1)$ is the entire output between the two red dots, $bigout(I_2)$ between the two blue dots, and $bigout(I_3)$ between the purple dots. All three blocks are non-empty, and $bigout(I_1 \cdot I_2 \cdot I_3)$ goes from the first red to the second purple dot. Black non-dashed arrows stand for **C**-small blocks. □

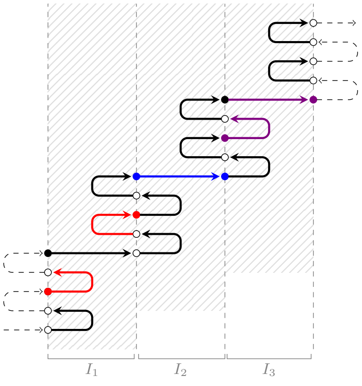

## 5 Proof of Theorem 1

This section is devoted to proving the characterization of one-way resynchronizability in the bounded-visit case. We will use the notion of *bounded-traversal* from [21], that was shown to characterize the class of bounded regular resynchronizers, in as much as bounded-delay characterizes rational resynchronizers [15].

**Definition 4 (traversal [21]).** *Let $\sigma = (u, v, orig)$ and $\sigma' = (u, v, orig')$ be two synchronized pairs with the same input and output words.*

*Given two input positions $y, y' \in \mathsf{dom}(u)$, we say that $y$* traverses *$y'$ if there is a pair $(y, z)$ of source and target origins associated with the same output position such that $y'$ is between $y$ and $z$, with $y' \neq z$ and possibly $y' = y$. More precisely:*

– *$(y, y')$ is a* left-to-right traversal *if $y \leq y'$ and for some output position $x$, $orig(x) = y$ and $z = orig'(x) > y'$;*
– *$(y, y')$ is a* right-to-left traversal *if $y \geq y'$ and for some output position $x$, $orig(x) = y$ and $z = orig'(x) < y'$.*

*A pair $(\sigma, \sigma')$ of synchronized pairs with input $u$ and output $v$ is said to have* $k$-bounded traversal*, with $k \in \mathbb{N}$, if every $y' \in \mathsf{dom}(u)$ is traversed by at most $k$ distinct positions of $\mathsf{dom}(u)$.*

*A resynchronizer $\mathcal{R}$ has* bounded traversal *if there is some $k \in \mathbb{N}$ such that every $(\sigma, \sigma') \in \mathcal{R}$ has $k$-bounded traversal.*

**Lemma 8 ([21]).** *A regular resynchronizer is bounded if and only if it has bounded traversal.*

*Proof (of Theorem 1).* First of all, observe that the implication $4 \to 1$ is straightforward. To prove the implication $1 \to 2$, assume that there is a $k$-bounded, regular resynchronizer $\mathcal{R}$ that is $T$-preserving and such that $\mathcal{R}(T)$ is order-preserving. Lemma 8 implies that $\mathcal{R}$ has $t$-bounded traversal, for some constant $t$. We head towards proving that $T$ has cross-width bounded by $t + k$. Consider two synchronized pairs $\sigma = (u, v, orig)$ and $\sigma' = (u, v, orig')$ such that $\sigma \in [\![T]\!]_o$ and $(\sigma, \sigma') \in \mathcal{R}$, and consider a cross $(X_1, X_2)$ of $\sigma$. We claim that $|orig(X_1)|$ or $|orig(X_2)|$ is at most $t + k$. Let $x_1 = \min(orig(X_1))$, $x'_1 = \max(orig'(X_1))$, $x_2 = \max(orig(X_1))$, and $x'_2 = \min(orig'(X_2))$. Since $(X_1, X_2)$ is a cross, we have $x_1 > x_2$, and since $\sigma'$ is order-preserving, we have $x'_1 \leq x'_2$. Now, if $x'_1 > x_2$, then at least $|orig(X_2)| - k$ input positions from $X_2$ traverse $x'_1$ to the right (the $-k$ term is due to the fact that at most $k$ input positions can be resynchronized to $x'_1$). Symmetrically, if $x'_1 \leq x_2$, then at least $|orig(X_1)| - k$ input positions from $X_1$ traverse $x_2$ to the left (the $-k$ term accounts for the case where some positions are resynchronized to $x'_1$ and $x'_1 = x_2$). This implies $\min(|orig(X_1)|, |orig(X_2)|) \leq t + k$, as claimed.

The remaining implications rely on the assumption that $T$ is bounded-visit.

The implication $2 \to 3$ is shown by contraposition: one considers a successful run $\rho$ with an inversion, and shows that crosses of arbitrary width emerge after pumping the loops of the inversion (here Lemma 3 is crucial).

The proof of $3 \to 4$ is more involved, we only sketch it here. Assuming that no successful run of $T$ has inversions we build a partially bijective, regular resynchronizer $\mathcal{R}$ that is $T$-preserving and $\mathcal{R}(T)$ is order-preserving. The resynchronizer $\mathcal{R}$ uses some parameters to guess a successful run $\rho$ of $T$ on $u$ and a factorization tree of bounded height for $\rho$. Formally, a *factorization tree* for a sequence $\alpha$ of monoid elements (e.g. the flows $\mathit{flow}_\rho([y,y])$ for all input positions $y$) is an ordered, unranked tree whose yield is the sequence $\alpha$. The leaves of the factorization tree are labeled with the elements of $\alpha$. All other nodes have at least two children and are labeled by the monoid product of the child labels (in our case by the flows of $\rho$ induced by the covered factors in the input). In addition, if a node has more than two children, then all its children must have the same label, representing an idempotent element of the monoid. By Simon's factorization theorem [23], every sequence of monoid elements has some factorization tree of height at most linear in the size of the monoid (in our case, at most $3|M_T|$, see e.g. [8]).

*Parameters.* We use input parameters to encode the successful run $\rho$ and a factorization tree for $\rho$ of height at most $H = 3|M_T|$. These parameters specify, for each input interval corresponding to a subtree, the start and end positions of the interval and the label of the root of the subtree. Correctness of these annotations can be enforced by an MSO sentence $\mathsf{ipar}$. The run and the factorization tree also need to be encoded over the output, using output parameters. More precisely, given a level in the tree and an output position, we need to be able to determine the flow and the productive edge that generated that position. We omit the technical details for checking correctness of the output annotation using the formulas $\mathsf{opar}$, $\mathsf{move}_\tau$ and $\mathsf{next}_{\tau,\tau'}$.

*Moving origins.* For each level $\ell$ of the factorization tree, a partial resynchronization relation $\mathcal{R}_\ell$ is defined. The relation is partial in the sense that some output positions may not have a source-target origin pair defined at a given level. But once a source-target pair is defined for some output position at a given level, it remains defined for all higher levels.

In the following we write $\mathit{bigout}(p)$ for the dominant output interval associated with the input interval $I(p)$ corresponding to a node $p$ in the tree. For every level $\ell$ of the factorization tree, the resynchronizer $\mathcal{R}_\ell$ will be a partial function from source origins to target origins, and will satisfy the following:

- the set of output positions for which $\mathcal{R}_\ell$ defines target origins is the union of the intervals $\mathit{bigout}(p)$ for all nodes $p$ at level $\ell$;
- $\mathcal{R}_\ell$ only moves origins within the same interval at level $\ell$, that is, $\mathcal{R}_\ell$ defines only pairs $(y,z)$ of source-target origins such that $y,z \in I(p)$ for some node $p$ at level $\ell$;
- the target origins defined by $\mathcal{R}_\ell$ are order-preserving within every interval at level $\ell$, that is, for all output positions $x < x'$, if $\mathcal{R}_\ell$ defines the target origins of $x, x'$ to be $z, z'$, respectively, and if $z, z' \in I(p)$ for some node $p$ at level $\ell$, then $z \le z'$;
- $\mathcal{R}_\ell$ is $\ell \cdot 4K\mathbf{C}$-bounded, namely, there are at most $\ell \cdot 4K\mathbf{C}$ distinct source origins that are moved by $\mathcal{R}_\ell$ to the same target origin.

The construction of $\mathcal{R}_\ell$ is by induction on $\ell$. For a binary node $p$ at level $\ell$ with children $p_1, p_2$, the resynchronizer $\mathcal{R}_\ell$ inherits the source-origin pairs from level $\ell - 1$ for output positions that belong to $bigout(p_1) \cup bigout(p_2)$. Note that $bigout(p_1) < bigout(p_2)$ by Lemma 5, so $\mathcal{R}_\ell$ is order-preserving inside $bigout(p_1) \cup bigout(p_2)$. Output positions inside $bigout(p) \setminus (bigout(p_1) \cup bigout(p_2))$ are moved in an order-preserving manner to one of the extremities of $I(p)$, or to the last position of $I(p_1)$. Boundedness of $\mathcal{R}_\ell$ is guaranteed by Lemma 6.

The case where $p$ is an idempotent node at level $\ell$ with children $p_1, p_2, \ldots, p_n$ follows a similar approach. For brevity, let $I_i = I(p_i)$ and $B_i = bigout(p_i)$, and observe that, by Lemma 5, $B_1 < B_2 < \cdots < B_n$. Lemma 7 provides a decomposition of $bigout(p)$ as $B_1 \cdot J_1 \cdot B_2 \cdot J_2 \cdot \ldots \cdot J_{n-1} \cdot B_n$, for some $2K\mathbf{C}$-small output intervals $J_k$ with origins inside $I_k \cup I_{k+1}$, for $k = 1, \ldots, n-1$. As before, the resynchronizer $\mathcal{R}_\ell$ behaves exactly as $\mathcal{R}_{\ell-1}$ for the output positions inside the $B_k$'s. For any other output position, say $x \in J_k$, the resynchronizer $\mathcal{R}_\ell$ will move the origin either to the last position of $I_k$ or to the first position of $I_{k+1}$, depending on whether the source origin of $x$ belongs to $I_k$ or $I_{k+1}$. □

## 6 Proof overview of Theorem 2

The main obstacle towards dropping the bounded-visit restriction from Theorem 1, while maintaining the effectiveness of the characterization, is the lack of a bound on the number of flows. Indeed, for a transducer $T$ that is not necessarily bounded-visit, there is no bound on the number of flows that encode successful runs of $T$, and thus the proofs of the implications $2 \to 3 \to 4$ are not applicable anymore. However, the proofs of the implications $1 \to 2$ and $4 \to 1$ remain valid, even for a transducer $T$ that is not bounded-visit.

The idea for proving Theorem 2 is to transform $T$ into an equivalent bounded-visit transducer $low(T)$, so that the property of one-way resynchronizability is preserved. More precisely, given a two-way transducer $T$, we construct:

1. a bounded-visit transducer $low(T)$ that is classically equivalent to $T$,
2. a 1-bounded, regular resynchronizer $\mathcal{R}$ that is $T$-preserving and such that $\mathcal{R}(T) =_o low(T)$.

We can apply our characterization of one-way resynchronizability in the bounded-visit case to the transducer $low(T)$. If $low(T)$ is one-way resynchronizable, then by Theorem 1 we obtain another partially bijective, regular resynchronizer $\mathcal{R}'$ that is $low(T)$-preserving and such that $\mathcal{R}'(low(T)))$ is order-preserving. Thanks to Lemma 2, the resynchronizers $\mathcal{R}$ and $\mathcal{R}'$ can be composed, so we conclude that the original transducer $T$ is one-way resynchronizable. Otherwise, if $low(T)$ is not one-way resynchronizable, we show that neither is $T$. This is precisely shown in the lemma below.

**Lemma 9.** *For all transducers $T, T'$, with $T'$ bounded-visit, and for every partially bijective, regular resynchronizer $\mathcal{R}$ that is $T$-preserving and such that*

*$\mathcal{R}(T) =_o T'$, $T$ is one-way resynchronizable if and only if $T'$ is one-way resynchronizable.*

There are however some challenges in the approach described above. First, as $T$ may output arbitrarily many symbols with origin in the same input position, and $low(T)$ is bounded-visit, we need $low(T)$ to be able to produce arbitrarily long outputs within a single transition. For this reason, we allow $low(T)$ to be a transducer with *regular outputs*. The transition relation of such a transducer consists of finitely many tuples of the form $(q, a, L, q')$, with $q, q' \in Q$, $a \in \Sigma$, and $L \subseteq \Gamma^*$ a regular language over the output alphabet. The semantics of a transition rule $(q, a, L, q')$ is that, upon reading $a$, the transducer can switch from state $q$ to state $q'$, and move its head accordingly, while outputting any word from $L$. We also need to use transducers with common guess. Both extensions, regular outputs and common guess, already appeared in prior works (cf. [5,7]), and the proof of Theorem 1 in the bounded-visit case can be easily adapted to these features.

There is still another problem: we cannot always expect that there exists a bounded-visit transducer $low(T)$ classically equivalent to $T$. Consider, for instance, the transducer that performs several passes on the input, and on each left-to-right pass, at an arbitrary input position, it copies as output the letter under its head. It is easy to see that the Parikh image of the output is an exact multiple of the Parikh image of the input, and standard pumping arguments show that no bounded-visit transducer can realize such a relation.

A solution to this second problem is as follows. Before trying to construct $low(T)$, we test whether $T$ satisfies the following condition on vertical loops (these are runs starting and ending at the same position and at the same state). There should exist some $K$ such that $T$ is *$K$-sparse*, meaning that the number of different origins of outputs generated inside some vertical loop is at most $K$. If this condition is not met, then we show that $T$ has unbounded cross-width, and hence, by the implication $1 \to 2$ of Theorem 1, $T$ is not one-way resynchronizable. Otherwise, if the condition holds, then we show that a bounded-visit transducer $low(T)$ equivalent to $T$ can indeed be constructed.

## 7 Complexity

We discuss the effectiveness and complexity of our characterization. For a $k$-visit transducer $T$, the effectiveness of the characterization relies on detecting inversions in successful runs of $T$. It is not difficult to see that this can be decided in space that is polynomial in the size of $T$ and the bound $k$. We can also show that one-way resynchronizability is PSPACE-hard. For this we recall that the emptiness problem for two-way finite automata is PSPACE-complete. Let $A$ be a two-way automaton accepting some language $L$, and let $\Sigma$ be a binary alphabet disjoint from that of $L$. The function $\{(w \cdot a_1 \dots a_n, a_n \dots a_1) \mid w \in L, a_1 \dots a_n \in \Sigma^*, n \geq 0\}$ can be realized by a two-way transducer $T$ of size polynomial in $|A|$, and $T$ is one-way resynchronizable if and only if $L$ is empty.

In the unrestricted case, we showed that one-way resynchronizability is decidable (Theorem 2). We briefly outline the complexity of the decision procedure:

1. First one checks that $T$ is $K$-sparse for some $K$. To do this, we construct from $T$ the regular language $L$ of all inputs with some positions marked that correspond to origins produced within the same vertical loop. Bounded sparsity is equivalent to having a uniform bound on the number of marked positions in every input from $L$. Standard techniques for two-way automata allow to decide this in space that is polynomial in the size of $T$. Moreover, this also gives us a computable exponential bound to the largest constant $K$ for which $T$ can be $K$-sparse.
2. Next, we construct from the $K$-sparse transducer $T$ a bounded-visit transducer $T'$ that is classically equivalent to $T$ and has exponential size.
3. Finally, we decide one-way resynchronizability of $T'$ by detecting inversions in successful runs of $T'$ (Theorem 1).

Summing up, one can decide one-way resynchronizability of unrestricted two-way transducers in exponential space. It is open if this bound is optimal. We also do not have any interesting bound on the size of the resynchronizer that witnesses one-way resynchronizability, both in the bounded-visit case and in the unrestricted case. Similarly, we lack upper and lower bounds on the size of the resynchronized one-way transducers, when these exist.

## 8 Conclusions

As the main contribution of this paper, we provided a characterization for the subclass of two-way transducers that are one-way resynchronizable, namely, that can be transformed by some bounded, regular resynchronizer, into an origin-equivalent one-way transducer.

There are similar definability problems that emerge in the origin semantics. For instance, one could ask whether a given two-way transducer can be resynchronized, through some bounded, regular resynchronization, to a relation that is origin-equivalent to a first-order transduction. This can be seen as a relaxation of the first-order definability problem in the origin semantics, namely, the problem of telling whether a two-way transducer is origin-equivalent to some first-order transduction, shown decidable in [4]. It is worth contrasting the latter problem with the challenging open problem whether a given transduction is equivalent to a first-order transduction in the classical setting.

*Acknowledgments.* We thank the FoSSaCS reviewers for their constructive and useful comments.
